# The interaction between domestic and outward foreign direct investment in China: The influence of region-specific context

**DOI:** 10.1371/journal.pone.0314109

**Published:** 2024-11-21

**Authors:** David W. H. Wong, Simon X. B. Zhao, Will W. Qiang, Harry F. Lee

**Affiliations:** 1 Department of Management, The Hang Seng University of Hong Kong, Hang Shin Link, Siu Lek Yuen, Shatin, New Territories, Hong Kong; 2 Faculty of Humanities and Social Sciences, BNU-HKBU United International College, Zhuhai, Guangdong Province, China; 3 Department of Geography and Resource Management, The Chinese University of Hong Kong, Shatin, New Territories, Hong Kong; University Putra Malaysia: Universiti Putra Malaysia, MALAYSIA

## Abstract

Since the Chinese government launched the Belt and Road Initiative, state-owned enterprises and private conglomerates have increased overseas investment. The massive outward foreign direct investment (OFDI) profoundly affects China’s regional development to varying degrees. Existing studies have mainly investigated the effect of foreign direct investment inflow on domestic investment (DI), but only a few studies have examined the impact of OFDI on DI. Though most researchers have indicated that OFDI activities can promote (or inhibit) DI in a particular country, they have overlooked how OFDI’s geographical distribution and motivation across the Chinese macro-regions have influenced DI. To fill these gaps, this paper examines the impact of such OFDI on DI in China and its macro-regions, using a province-level dataset from 2005 to 2021. It employs panel regression and rigorously tests the data using the generalised method of moments to address endogeneity issues. We also investigate the investment motivations of the top 100 Chinese multinationals with significant FDI outflows from 2015 to 2018. We find that OFDI has positively and markedly influenced DI in eastern coastal and central inland regions but not in western China. Though OFDI has positively affected DI in China, it may not hold in a regional context. Such OFDI seeks to enhance the efficiency of existing investment, expand domestic markets, and look for strategic assets. Our findings indicate that central and provincial governments could develop OFDI policies to cater to significant regional variations. Local governments should also consider the various OFDI motivations of Chinese multinationals and provide adequate support for developing and implementing local investment policies that encourage domestic firms to invest abroad.

## 1. Introduction

Since the late 1970s, China’s opening-up policy and economic reforms have encouraged the development of outward foreign direct investment (OFDI) policies. Many researchers have described the gradual, staged implementation of these policies [1,2]. From 1992 to 2001, the Chinese central government encouraged companies engaged in global trade and cross-border production networks to invest in foreign markets to support export and manufacturing operations abroad. Following China’s accession to the World Trade Organization in 2001, the Chinese government adopted its *Going Out* (*zou chu qu*) strategy. This *Going Out* policy encouraged Chinese firms to seek new markets. Since 2013, the Chinese government has implemented a new global development strategy. Belt and Road Initiatives sparked interest in major overseas projects in finance, mining, wholesale and retail, manufacturing, and transportation services [3]. By August 2023, the Chinese government signed cooperation memoranda or issued joint statements with 152 countries and 32 international organisations, partly building out the Belt and Road Initiatives. With the progressive internationalisation of the Chinese yuan (RMB), the rapid growth of digital financial technology, and the profound influence of the host country’s governance, OFDI have increased dramatically in China over the last decade [4–6].

From an academic perspective, previous literature has examined chiefly the influence of China’s OFDI on economic growth and development [7,8]. Still, few studies have investigated the nexus between OFDI and domestic investment (DI) in regions (or provinces). Since China’s growth model heavily relies on domestic capital formation, recent significant capital outflows have alarmed regional planners and policymakers. These policymakers worry about their potential effects on DI [9]. China became the world’s first largest foreign direct investment (FDI) outflow source in 2020, totalling roughly US$ 133 billion [10]. The size of these FDI outflows matters less than how they compare to the flow of DI. On the one hand, OFDI and DI are complements [11]. Both serve to establish backward and forward links in the production chains operating in China. On the other hand, such OFDI displaces DI for foreign activities [12]. As firms shift a portion of their production abroad, the financial resources available for domestic development decline. A consensus on the effect of OFDI on domestic gross capital formation in China remains elusive.

Studies of foreign countries have made greater headway. Many researchers have looked at OFDI and DI in countries like the United States, Germany, Italy, and newly industrialised countries like South Korea [13,14]. Existing studies examine the firm level [15], industry level [16], and country level [17], respectively. Some studies cover a limited period, a few industries, or only multinational enterprises [18]. These limited studies thus likely suffer from sample selection bias. Additionally, burgeoning literature examines OFDI-DI association across countries within different continents (or economic blocs), but there is a lack of studies investigating the above relationship *within* a particular country. From a geographic perspective, many researchers treat China as a single spatial aggregate and generalise the findings to the entire country. Yet, subject to the significant differences in factor endowments, political and economic structures, and socio-cultural backgrounds between Chinese regions and Belt and Road Initiatives projects abroad, those previous studies that treated China as a homogeneous entity (i.e., without considering the vast differences between inland and coastal areas) may somehow constrain their applicability [19,20]. Apart from the previous literature focusing on China’s OFDI-DI nexus, a paucity of studies has examined how OFDI’s motivation has influenced DI across the Chinese macro-regions [21].

Against this backdrop, three related questions remain unsolved: (1) At the macroeconomic level, what is the regional impact of the OFDI on DI in developing and emerging economies like China? (2) How do OFDI motivations influence China’s and its macro-regions’ DI? (3) More importantly, what are the policy implications for improving OFDI and DI’s efficiency? We use the panel regression method on annual data at the provincial level from 2005 to 2021 to investigate the OFDI-DI nexus for China and its macro-regions.

This research is a pioneering study, contributing three ways to the academic debate on the OFDI-DI association. First, unlike previous studies, we show the precise impacts of the OFDI on DI for China and its macro-regions rather than the aggregated effect of OFDI on DI. Second, contrasted with earlier studies debating the OFDI-DI nexus at the country level, we find that the OFDI for China, Eastern, and Central China has a complementary relationship with their DI. At the same time, that for Western China does not affect DI. The diverse results indicate that regional factors matter. Differences in institutional quality, financial capabilities, and levels of economic development between coastal and inland regions help account for these differing relationships. Second, we describe the motivations for engaging in OFDI. The reasons for OFDI are based on improving DI efficiency, acquiring natural resources, or buying strategic assets [21]. Our findings intimate that much OFDI seeks to enhance the efficiency of existing investment, expand overseas markets, and search for strategic assets. Chinese policymakers would thus do well in designing policies targeted at the motivations and aims of such OFDI at the regional/provincial level.

The rest of the research is organised as follows. The following section critically reviews the literature on the OFDI-DI nexus. The second section presents data and methodology. Section 3 shows our regression results and provides an in-depth analysis of them. Finally, Section 4 concludes with a summary of our findings and policy implications.

## 2. Theoretical framework and literature

### 2.1. The relationship between OFDI and DI: Theories and empirical evidence

Scholars have debated the relationship between OFDI and DI over decades. From a theoretical perspective, the mechanism of OFDI’s influence over the home country’s DI mainly arises through financial and product markets. In an imperfect financial market, FDI outflows may raise domestic interest rates by transferring funds abroad, making it difficult for domestic firms to borrow money. Likewise, local firms seeking to invest in foreign markets may decrease domestic exports by funding overseas manufacturing operations and diverting DI. Consequently, OFDI leads to replace DI [22]. Nevertheless, if OFDI augments the home country’s exports through forward and backward linkages, it may complement DI. Thus, as FDI outflows may exert positive, negative, or neutral influence over the home country’s DI, Al-Sadig suggests reassessing the OFDI-DI nexus is necessary to determine the overall effects of such FDI outflows [23].

Several studies document different views on the OFDI-DI association. First, some researchers find OFDI leads to complement DI (i.e., crowding-in effect). As Desai, Foley, and Hines argue [19], looking specifically at the United States, outward FDI enabled U.S. parent companies to import lower-cost raw materials from foreign affiliates. Such FDI outflows also allowed these companies to export the intermediate inputs needed by their foreign affiliates. Industries including foreign affiliates in their value chains could reduce production costs and generate economies of scale, thus increasing domestic output and investment. As a result, the entire domestic economy can benefit from backward and forward-looking production links between local firms and multinational enterprises. In Taiwan, the study by Hsu, Wang, and Clegg supports a complementary relationship between FDI and gross capital formation has been found in Taiwan [11]. FDI helped allocate capital effectively between host and home regions, benefiting both areas. Taiwanese multinational corporations’ investment in China’s labour-intensive industrial value chains helped lower production costs. Similarly, Gondim, Ogasavara, and Masiero indicate FDI outflows from Brazil contributed to more local investment there. As such, OFDI is a crowded-in investment [24].

Second, in many developed economies, direct investment abroad has overtaken domestic investment [25]. In the analysis of OFDI and investment trends in 121 developing and emerging economies, FDI outflows serve to replace DI (i.e., crowding-out effect). Domestic bottlenecks and distortions in the home country’s economy, such as imperfect financial markets or capital controls, could help explain such negative associations. OFDI could quickly outstrip DI as many multinational enterprises in developing countries invested in foreign assets to collateralise domestic debts rather than diversify or mitigate financial risk. Supported by Sauramo, OFDI tempered DI activity in Finland. Such foreign direct investment out of Finland replaced Finnish exports abroad, dampening the demand for local labour. A lack of capital and an inefficient financial market also constitute the substitution effect of OFDI on DI. Finland’s strong OFDI growth primarily explains its multinational enterprises’ small local investment [26].

Third, some researchers have found the opposite results. They indicate the mixed relationship between FDI outflows and DI. In analysing Korean and Chinese OFDI from 1988 to 2002, only Chinese OFDI fluctuated with DI [27]. Even though incentives to engage in OFDI could depress DI in many developing countries, they had the opposite effect in some emerging economies. Cross-border FDI encouraged long-term investment between Southeast Asian countries. Such a result stemmed from the increased intra- and inter-firm trade such investment occasioned. Differing levels of technological advancement, institutional frameworks, and national development could also explain much variation between FDI and investment across ASEAN countries [28]. Moreover, the results were mixed when Kurtovic, Maxhuni, Halili, and Krasniqi examined the OFDI-DI relationship for the Central, East, and Southeast European countries. OFDI of Estonia and Lativa exert a crowding-in effect on DI, while those of Bulgaria, Poland, and Slovenia demonstrate a substitution effect on DI. Different motives, strategies, and decisions of domestic companies from the Central, East, and Southeast European countries display various specific features of their OFDI [29].

In Germany, OFDI triggered more DI in the short run but substituted for such investment in the longer run. High and rising labour costs and increased economic integration across the European Union sent several German multinational corporations into lower-cost Central and Eastern Europe manufacturing environments [14]. Previous research had an over-emphasised role of multinational investment in determining the extent of complementary or substitute domestic investment. They argue, instead, for host-country and industry-specific effects in driving OFDI-DI outcomes [11]. Yet, among these studies, Hejazi and Pauly argue that multinational enterprises’ production abroad, which gives rise to a positive, negative, or neutral effect on DI, hinges on the motivation behind OFDI [21].

### 2.2. OFDI motivations for DI

According to Dunning and Lauren, understanding OFDI is closely related to the eclectic paradigm, emphasising three important aspects: ownership-specific (O), location-specific (L), and internalisation-specific (I) [30]. The OLI paradigm postulates a firm will establish an offshore production base when a host country offers L-specific advantages, such as abundant raw materials, cheap labour, and huge market potential, to a firm possessing O-specific advantages, for instance, renowned brand name, and advanced technology and both advantages can be operated through internalisation of production through FDI. Hence, each firm seeks the optimisation of O-, L-, and I-specific advantages.

Extended by the OLI paradigm, four motivations for multinational enterprises to invest abroad have been described, thus affecting domestically more or less [21]. The *resource-seeking* multinational enterprises essentially choose to acquire natural resources unavailable in their home countries or at a lower cost. Such motivation would positively impact DI by crowding in work on primary commodity processing and intermediate consumption [24]. The *market-seeking* multinational enterprises might invest in substitutes for the country’s exports or relocate domestic production abroad in a trade bloc like the United States-Mexico-Canada Agreement region. A US firm investing in Mexico to sell to its roughly 130 consumers more directly might represent this motivation. The *efficiency-seeking* multinational enterprises might take advantage of factor price differences by investing in locations with lower production costs [21]. The *strategic assets*-seeking multinational enterprises might invest abroad to acquire complementary assets that bolster its competitive advantages [7]. However, some researchers have criticised these four motivations, failing to explain how these motivations work with the OFDI-DI nexus at the industry or firm levels because different OFDI motivations may produce different results (i.e., crowding-in or crowding-out effects) on DI [15].

### 2.3. China’s OFDI effects on DI–A normal or deviant case?

The findings on China similarly conflict with each other. Based on Ameer, Xu, and Alotaish, a macro-level study of OFDI on DI failed to find any cause-and-effect relationship between them in the short run. The positive association between the two ran only one way in the long run. China’s underdeveloped financial markets encouraged OFDI, as Chinese multinationals faced far tighter financial constraints than multinationals from advanced countries [31]. State-owned enterprises accounted for much of the Chinese multinationals’ OFDI, as they benefited from access to China’s rapidly accumulating foreign exchange reserves. Chinese overseas investment had little impact on local financial liquidity, and such OFDI did not replace domestic capital formation as a source of growth [7]. Though the analysis of FDI and DI considered factors contributing to domestic credit to China’s private sector, the real interest rate, and institutional quality, Chinese investors preferred to invest abroad rather than at home. Nevertheless, these authors overlooked the regional variation in OFDI and DI between Chinese regions. Some researchers have speculated that these studies’ neglect of a regional-level analysis may explain these seemingly contrasting results [12,32].

## 3. Methods and data

### 3.1. Data and variable selection

To address these gaps in the literature, our study uses a panel dataset spanning 31 Chinese provinces, municipalities, and autonomous regions from 2005 to 2021. The data come from various *China Statistical Yearbooks*, *China Provincial Statistical Yearbooks*, and *Chinese Outward Foreign Direct Investment Statistical Bulletins*. We break our data into three macro-regions–Eastern, Central, and Western China following the Seventh Five-Year Plan (1986–90) adopted by the Chinese government. Several provinces, municipalities, or autonomous regions with similar characteristics are aggregated into macro-regions. This regional delineation has been widely applied in academic research. Besides, we follow this grouping to conduct our inter-regional analysis to facilitate the comparison of estimation results with previous studies [7,8,33]. In this study, DI serves as our dependent variable.

We deploy two conceptual frameworks to inform our empirical methods. First, following Feldstein, Desai, Foley and Hines, and Ali, Wang, Morales, and Wang, we adopted an extension of Feldstein and Horioka’s model [16,18,25,34], where we assume that the level of DI depends on OFDI, inward foreign direct investment (IFDI), and domestic savings (DS) that are widely adopted by previous studies. Furthermore, as He, Wei and Xie, and Wei note, China’s regional development was sharply transformed by the fundamental processes of exerting globalising forces, the infusion of market mechanisms, and the decentralised control of economic development by local states over decades [35,36]. As such, the second model sees regional DI as the result of globalisation, marketisation, and the decentralised control of local economic development. We set up the model specification [i.e., Eq (1)]. Variable definitions, measurements, and data sources are presented in Table 1.

**Table 1:** Variables employed in this study and their measurements and data sources.

Variable	Measurement	Data source
DI	Domestic investment, measured by total investment in fixed assets invested by private enterprises over gross regional product	China Provincial and Municipal Statistical Yearbook
OFDI	Outward foreign direct investment, measured by outward direct investment flows divided by gross regional product	The Statistical Bulletin of China’s Outward Foreign Direct Investment
IFDI	Inward foreign direct investment, defined by inbound foreign direct investment flows divided by gross regional product	China Provincial and Municipal Statistical Yearbook
DS	Domestic savings, measured by the deposits held by financial intermediaries over gross regional product	China Provincial and Municipal Statistical Yearbook
PGR	Population growth, defined by the annual growth rate of the *de-facto* population at year-end	China Statistical Yearbook
EDUL	Education level, measured by the number of people who have completed at least secondary education divided by the total number of *de-facto* population at year-end	China Statistical Yearbook
RD	Research and development intensity, defined by research and development expenditure as a share of gross regional product	China Statistical Yearbook
EXPT	Export, defined by the total amount of the region’s exports to the host country over gross regional product	China Statistical Yearbook
INF	Investment in infrastructure, defined by gross fixed capital formation over gross regional product	China Statistical Yearbook
IND	Level of industrial development, measured by industrial added value divided by gross regional product	China Statistical Yearbook

### 3.2. Dependent and major explanatory variables

We use the share of total private enterprises’ fixed asset investment divided by gross regional product to gauge the impact of FDI on Chinese regional DI [19,23]. Moreover, we deploy OFDI flows divided by gross regional product as our major explanatory variable [13,23]. We do not take a priori stance on whether the relationship between FDI and regional DI should be positive or negative.

We include IFDI because some research finds a strong association with DI. IFDI boosts DI for firms seeking greater export competitiveness from economies of scale and agglomeration economies [37]. As inconclusive findings exist as to the direction of this relationship [38], we use a two-sided hypothesis (testing for a positive or negative relationship) between IFDI and DI. Such foreign-sourced investment could either crowd in or crowd out DI. Following the previous finding [25], we posit a positive relationship between DS and DI.

### 3.3. Control variables

Several other variables could interfere with the relationship between OFDI and DI. Population growth (PGR), educational level (EDUL), and research and development spending (RD) control for the influence of marketisation across Chinese regions. First, many countries are in various stages of demographic transition, affecting population dynamics. As such, we use the population growth rate (PGR) to control the influence of *de-facto* population [39]. Second, some research deploys the number of graduates who attain the highest levels of schooling (i.e., senior secondary school graduates) to proxy for labour quality. Thus, we apply the number of people who have completed at least secondary education (EDUL) to control for the educational level [40]. Third, following previous research, we choose research and development expenditure as a share of gross regional product (RD) to control research and development intensity [41]. Finally, we include each region’s export performance (EXPT) to control for broader globalisation affecting each region. To mitigate the effects of decentralisation, we use the variables level of industrial development (IND) and infrastructure investment (INF) [35].

### 3.4. Model specification

Our empirical model investigates the potential impact of OFDI on DI across China and three macro-regions. Basing our description of these macro-regions on China’s Seventh Five-Year Plan (1986–1990), Eastern China comprises Liaoning, Hebei, Beijing, Tianjin, Shandong, Jiangsu, Shanghai, Zhejiang, Fujian, Guangdong, Guangxi, and Hainan. Central China covers Heilongjiang, Jilin, Inner Mongolia, Shanxi, Henan, Anhui, Hubei, Jiangxi, and Hunan. Western China consists of Xinjiang, Gansu, Qinghai, Ningxia, Shaanxi, Tibet, Sichuan, Chongqing, Guizhou, and Yunnan.

We augment pooled ordinary least squares (OLS) regression with fixed-effects (FE) and random-effects (RE) models to test and potentially control for province- and time-specific effects. Our panel included observations on the above variables from 2005 to 2021. Eq (1) describes our regression model. The subscript *i* represents the 31 individual provinces, and *t* represents the 17 time periods. The parameter *α* represents the intercept, while *ε*_*it*_ is the error term for each regression.


$$DI_{it}=\alpha_{i}+\beta_{1}OFDI_{it}+\beta_{2}IFDI_{it}+\beta_{3}DS_{it}+\beta_{4}PGR_{it}+\beta_{5}EDUL_{it}+\beta_{6}RD_{it}+\beta_{7}EXPT_{it}+\beta_{8}INF_{it}+\beta_{9}IND_{it}+\varepsilon_{it}$$ (1)

Prior to estimation, the possibility of cross-sectional dependence in our model’s residuals must be investigated using the cross-sectional dependency (CSD) test proposed by Pesaran [42]. Cross-sectional dependency is defined as the correlation between cross-sectional units in panel data. Suppose the cross-sectional units (i.e., provinces) that comprise the time series are interdependent. In that case, any external shock, such as political, economic, social, and cultural associations, occurring in any unit in the time series will have a distinct influence on all units in the series. Otherwise, an external shock in one cross-sectional unit of the series within the panel will have an equal impact on all units, rendering the time series independent. Three CSD tests are used: The Breusch-Pagan Lagrange Multiplier (LM) [43], the Pesaran Scaled LM [44], and the Pesaran CD [44]. The null hypothesis for all three tests is no cross-sectional dependence. If the p-values exceed the 5% significance level, we will accept no cross-sectional dependence in the residuals.

After detecting cross-sectional dependency in our model, we use the slope homogeneity test to determine whether differences in one region’s system affect another, such as different socioeconomic structures. If the socioeconomic structure of one region differs from another, the slope coefficients are likely to be different. Otherwise, the slope coefficients may be homogeneous if the socioeconomic structures are comparable. To determine whether the slope coefficients of Eq (1) in cross-section are homogeneous, we use Hsiao’s [45] homogeneity test. Hsiao’s test examines three hypotheses: H_1_, H_2_, and H_3_. H_1_ states that the slope coefficients are homogeneous, whereas the alternative hypothesis allows for heterogeneity. While H_2_ adheres to the homogeneous assumption, the alternative H_2_ accepts heterogeneity. The final hypothesis, H_3_, states that the slope coefficients are homogeneous, whereas the alternative hypothesis claims they are partially homogeneous. If the p-values from the preceding tests are less than 0.05, the null hypotheses are rejected, indicating that the slope coefficients are heterogeneous.

Besides, we perform a robustness check by taking out the variable EXPT in the model specification [see Eq (1)] and examining whether the coefficients of OFDI are unbiased and consistent for China and its macro-regions. Lastly, in Eq (1), a potential causality exists between FDI and EXPT. FDI may contribute to export growth by facilitating technology transfers, upgrading the export structure of a host region, and increasing access to global distribution networks to sell products in international markets [46]. Conversely, expanding exports in the home regions lead to greater demand for good transportation infrastructure, which attracts more FDI. Additionally, historical events and activities may have a significant contemporaneous effect on DI. To address potential endogeneity issues, we use a panel generalised method of moments (GMM) procedure to confirm the dynamic nature of the model specification and run over-identification and serial correlation tests [47,48]. Because FDI may have lag effects on exports and *vice versa*, we also instrument FDI and EXPT variables with one-year lagged values.

As previously stated, we must perform the GMM procedure to validate our results. To begin, we run the OLS with a one-year lagged DI value to assess its persistence effect. If the DI’s one-year lagged coefficient is positive and significant at the 5 per cent level, the DI’s persistence effect is valid for the following tests. Second, to perform an overidentification test, we obtain the Hansen J-statistic for China and its macro-regions to determine whether all overidentification restrictions are valid. When the p-value falls between 0.25 and 1.00 [49], we accept the null hypothesis, indicating that instrument validity has been established. Otherwise, we will reject the null hypothesis due to at least one invalid instrument [50]. Finally, to perform the serial correlation test, we calculate the value of the second-order autocorrelation AR(2). We propose the null hypothesis of no second-order serial correlation. If the p-value of AR(2) is greater than 0.05, we do not reject the null hypothesis, indicating that the moment conditions are correctly specified.

### 3.5. Assessment of the impact of OFDI motivations on DI

Likewise, we speculate about the motivations of large Chinese MNEs to invest abroad directly. To study their motivations, we identify the top 100 Chinese multinationals, ranked by their stocks of OFDI in 2015–2018, as reported in the *Outward Foreign Direct Investment Statistics Bulletin of China*, and *Report on Development of China’s Outward Investment*. We examine recent merger and acquisition transaction information from financial intelligence, annual reports, and corporate announcements. After investigating the details of relevant merger and acquisition transactions, we encapsulate the motivations for OFDI of those companies in the respective macro-regions. We code their experiences as either resource-seeking, market-seeking, efficiency-seeking, or strategic-assets-seeking OFDI as recorded in the *Outward Foreign Direct Investment Statistics Bulletin of China* and *Report on Development of China’s Outward Investment*. We are attempting to determine how OFDI motivation affects DI in conjunction with the statistical findings.

## 4. Estimation results and discussion

### 4.1. Descriptive statistics

Before analysing our results, we need to examine the descriptive statistics of each variable. Table 2 indicates the descriptive statistics, whereas Table 3 depicts the correlation matrix of the variables employed in this study. Our statistical results show that almost all variables are positively correlated, and no apparent outliers have been revealed. Moreover, no multicollinearity among our variables arises as no correlation coefficients appear sufficiently high.

**Table 2:** Descriptive statistics of the variables employed in this study.

Variables	DI	OFDI	IFDI	DS	PGR	EDUL	RD	EXPT	INF	IND
Mean	0.691	0.004	0.022	1.754	0.006	0.006	0.013	0.149	0.686	0.080
Median	0.688	0.002	0.018	1.586	0.004	0.006	0.011	0.076	0.533	0.072
Maximum	1.504	0.071	0.104	5.587	0.058	0.009	0.061	0.904	54.346	0.204
Minimum	0.181	0.003	0.000	0.563	0.051	0.002	0.000	0.004	0.243	-0.178
Standard deviation	0.265	0.007	0.018	0.777	0.012	0.001	0.010	0.169	2.349	0.039
Skewness	0.301	4.768	1.308	2.363	0.869	-0.365	2.386	2.104	22.737	-0.061
Kurtosis	0.191	29.671	1.970	6.802	4.155	0.463	7.957	4.410	520.258	3.482
Observation	527	527	527	527	527	527	527	527	527	527

**Table 3:** Correlation matrix of the variables employed in this study.

Variables	DI	OFDI	IFDI	DS	PGR	EDUL	RD	EXPT	INF
OFDI	0.013***								
IFDI	0.110***	0.015***							
DS	0.029***	0.196***	0.007**						
PGR	0.015***	0.003	0.055***	0.069***					
EDUL	0.208***	0.112***	0.047***	0.202***	0.009**				
RD	0.144***	0.101***	0.158***	0.262***	0.106***	0.122***			
EXPT	0.287***	0.033***	0.211***	0.056***	0.213***	0.123***	0.219***		
INF	-0.001	-0.002	-0.002	-0.001	-0.002	-0.002	0.005*	0.002	
IND	0.090***	-0.001	0.068***	0.058***	0.017***	-0.002	0.061***	0.082***	0.004*

*Note*: The symbols *, ** and ***denote statistical significance at the 10%, 5%, and 1%, respectively (* *p* < 0.10, ** *p* < 0.05, and *** *p* < 0.01).

### 4.2 Cross-sectional dependence and slope homogeneity

As previously mentioned, testing cross-sectional dependency and slope homogeneity in the panel data in the study of panel data regression is essential for choosing the appropriate estimators. Hence, we run the cross-sectional dependency and slope homogeneity tests, and the results are presented in Table 4. For cross-sectional dependence, the CD’s test statistic in Eastern China is significant at a 5 per cent level, while the rest of CD_*BP*_’s, CD_*PS*_’s and CD’s test statistic at the country and regional levels are significant at a 1 per cent level, implying that all null hypotheses (i.e., H_1_, H_2_ and H_3_) are rejected. Our results support the fact that cross-sectional dependency exists across provinces. In slope homogeneity, as our results demonstrated that the p-values for all H_1_, H_2_ and H_3_ in the national and regional context are less than 0.05, all null hypotheses are rejected, showing that the slope of coefficients is heterogeneous. Briefly, cross-sectional dependence and slope heterogeneity exist in the panel data of China, Eastern China, Central China, and Western China. According to Sarafidis and Robertson [51], using FE models can control province-specific effects, thereby capturing time-invariant heterogeneity across provinces and mitigating the bias of these FE estimators caused by cross-sectional dependence in panel data. Hence, FE models are primarily considered in our regression models, while RE models are also run for reference.

**Table 4:** Cross-sectional dependence and slope homogenous tests of China and its macro-regions.

Test/ regions	Test statistics			
Cross-sectional dependency test	China	Eastern China	Central China	Western China
CD_*BP*_	3,268.272***	416.526***	98.931***	177.147***
CD_*PS*_	91.923***	30.509***	7.417***	13.929***
CD	34.692***	2.298**	3.119***	6.913***
**Slope homogeneous test (Hypotheses)**				
H_1_	31.598***	48.348***	9.334***	8.931***
H_2_	18.622***	42.761***	5.074***	5.741***
H_3_	13.778***	4.181***	14.881***	10.624***

*Note*: CD_*BP*_ represents the cross-sectional dependence test of Breusch and Pagan [43], whereas CD_*PS*_ and CD are those of Pesaran [44], respectively; The symbols *, ** and *** denote statistical significance at the 10%, 5% and 1%, respectively (* *p* < 0.10, ** *p* < 0.05 and *** *p* < 0.01).

### 4.3. Regression results

Table 5 provides a first look at our variables for China. DI has a statistically significant relationship with OFDI. Reassuringly, DS also statistically significantly vary with DI. With the control variables included, our FE models have relatively good explanatory power with R-squared values of over 0.6 across models. Also, our Hausman test results demonstrate that FE models stratified by provinces and controlling for endogeneity are more appropriate for the data.

**Table 5:** Regression results for China nationwide.

Dependent variable	Domestic investment (DI)				
	Model (1)–OLS	Model (2)–RE	Model (3)–FE (main specification)	Model (4)–FE (robustness check)	Model (5)–FE (GMM)
Intercept	0.539*** (0.067)	0.209*** (0.067)	-0.095 (0.074)	-0.101 (0.072)	0.038 (0.058)
OFDI	2.765** (1.369)	3.161*** (1.196)	3.267** (1.267)	3.363** (1.233)	2.197** (0.993)
IFDI	-0.636 (0.577)	0.016 (0.613)	0.659 (0.678)	0.599 (0.652)	0.946* (0.506)
DS	0.001 (0.017)	0.084*** (0.019)	0.234*** (0.026)	0.237*** (0.025)	0.143*** (0.031)
PGR	3.123*** (0.836)	2.055*** (0.748)	2.023** (0.785)	1.979** (0.773)	1.832*** (0.618)
EDUL	61.484*** (7.769)	81.855*** (7.576)	61.025*** (8.719)	60.361*** (8.482)	29.824*** (8.738)
RD	-2.737** (1.268)	1.568 (1.303)	6.258*** (1.471)	6.288*** (1.466)	4.071*** (1.205)
EXPT	-0.617*** (0.071)	-0.569*** (0.091)	-0.043 (0.131)	- -	-0.033 (0.099)
INF	-0.002 (0.004)	-0.001 (0.003)	-0.001 (0.003)	-0.001 (0.003)	-0.001 (0.002)
IND	-1.136*** (0.268)	-1.231*** (0.232)	-1.161*** (0.239)	-1.162*** (0.239)	-0.978*** (0.245)
Observations	527	527	527	527	527
R-squared	0.421	0.285	0.667	0.668	0.836
Hausman Test (p-value)			106.897 (0.000)	109.083 (0.000)	

*Note*: The symbols *, ** and *** denote statistical significance at the 10%, 5% and 1%, respectively (* *p* < 0.10, ** *p* < 0.05 and *** *p* < 0.01); Parentheses state standard deviation.

Running the same regressions for China’s eastern provinces shows somewhat different trends. Table 6 shows that, for these provinces, OFDI clearly explained DI over time. All models produced statistically significant results. Based on the Hausman test result, we choose the RE model. Yet, our finding indicates the parameter estimates for IFDI proved highly unstable. According to the OLS model, IFDI tends to replace DI. IFDI appears to continue to have a significant relationship with DI when controlling province-level effects.

**Table 6:** Regression results for Eastern China.

Dependent variable	Domestic investment (DI)				
	Model (6)–OLS	Model (7)–RE	Model (8)–FE (main specification)	Model (9)–FE (robustness check)	Model (10)–FE (GMM)
Intercept	0.671*** (0.109)	0.668*** (0.109)	0.798*** (0.049)	0.108 (0.137)	0.305** (0.151)
OFDI	2.492** (1.218)	2.381* (1.222)	1.843** (0.743)	2.379* (1.349)	2.223* (1.355)
IFDI	-1.341** (0.625)	-1.261** (0.626)	-1.237*** (0.426)	-0.936 (0.911)	0.161 (1.008)
DS	-0.083*** (0.021)	-0.083*** (0.021)	-0.105*** (0.009)	0.092** (0.041)	0.054 (0.044)
PGR	1.422 (0.952)	1.462 (0.953)	1.579*** (0.505)	0.933 (1.001)	1.432 (1.116)
EDUL	34.205** (14.078)	34.778*** (14.024)	9.464 (7.008)	28.035* (15.163)	23.156 (18.084)
RD	1.595 (1.408)	1.594 (1.407)	1.759*** (0.575)	4.333*** (1.578)	3.806** (1.589)
EXPT	-0.585*** (0.066)	-0.581*** (0.066)	-0.524*** (0.042)	- -	-0.267 (0.176)
INF	-0.001 (0.003)	-0.001 (0.003)	0.001 (0.003)	-0.001 (0.003)	-0.001 (0.003)
IND	-0.024 (0.459)	-0.046 (0.461)	-0.011 (0.222)	0.218 (0.537)	-0.299 (0.568)
Observations	204	204	204	204	204
R-squared	0.511	0.532	0.728	0.582	0.609
Hausman Test (p-value)			45.193 (0.000)	18.435 (0.018)	

*Note*: The symbols *, ** and *** denote statistical significance at the 10%, 5% and 1%, respectively (* *p* < 0.10, ** *p* < 0.05 and *** *p* < 0.01). Parentheses state standard deviation.

The results for China’s central provinces confirm the general patterns described for the whole sample. Table 7 shows these results. OFDI also statistically significantly varies with DI across models. Only the OLS model shows a statistically significant relationship between DI and IFDI. Such a finding shows the fragility of any conclusion about IFDI and DI. Treating central and coastal provinces separately increases our models’ explanatory power, as the R-squared values for these models are like those in Table 5. Given that FE modelling should have controlled for province-level effects, these results indicate that some pan-provincial effects across China’s central provinces appear to have some effects. Our literature review did not mention any specific regional factor affecting these central provinces. These relationships break down for Western China. Table 8 exhibits the lack of statistically significant OFDI coefficients for these provinces only. High R-squared values suggest the absence of a relationship does not stem from model misspecification or other problems.

**Table 7:** Main empirical results for Central China.

Dependent variable	Domestic investment (DI)				
	Model (11)–OLS	Model (12)–RE	Model (13)–FE (main specification)	Model (14)–FE (robustness check)	Model (15)–FE (GMM)
Intercept	-0.362*** (0.116)	-0.321*** (0.109)	-0.325*** (0.097)	-0.234** (0.097)	-0.219** (0.091)
OFDI	36.723*** (8.683)	23.311*** (7.069)	21.661*** (6.695)	22.027*** (6.948)	12.553** (6.528)
IFDI	5.072*** (1.572)	-0.191 (1.759)	-1.152 (1.764)	-0.178 (1.806)	-1.278 (1.682)
DS	0.081** (0.036)	0.167*** (0.033)	0.177*** (0.032)	0.175*** (0.033)	0.113*** (0.037)
PGR	-0.232 (1.384)	-2.586** (1.211)	-2.647** (1.153)	-2.083* (1.184)	-1.673 (1.011)
EDUL	1.848 (13.436)	-19.329 (12.671)	-19.775 (12.139)	-21.185* (12.591)	-14.914 (11.176)
RD	16.857*** (3.603)	29.772*** (4.271)	31.345*** (4.172)	34.017*** (4.251)	24.288*** (5.377)
EXPT	1.404** (0.601)	1.671*** (0.521)	1.676*** (0.495)	- -	1.297*** (0.448)
INF	0.965*** (0.111)	0.909*** (0.108)	0.915*** (0.106)	0.811*** (0.105)	0.705*** (0.118)
IND	-0.243 (0.377)	-0.409 (0.302)	-0.452 (0.286)	-0.125 (0.279)	-0.511* (0.271)
Observations	153	153	153	153	153
R-squared	0.563	0.722	0.791	0.775	0.853
Hausman Test (p-value)			222.234 (0.000)	152.398 (0.000)	

*Note*: The symbols *, **, and *** denote statistical significance at the 10%, 5% and 1%, respectively (* *p* < 0.10, ** *p* < 0.05 and *** *p* < 0.01). Parentheses state standard deviation.

**Table 8:** Main empirical results for Western China.

Dependent variable	Domestic investment (DI)				
	Model (16)–OLS	Model (17)–RE	Model (18)–FE (main specification)	Model (19)–FE (robustness check)	Model (20)–FE (GMM)
Intercept	-0.185** (0.081)	-0.185** (0.066)	0.319*** (0.106)	0.306*** (0.105)	-0.295 (0.153)
OFDI	4.582* (2.612)	4.583** (2.104)	2.254 (2.319)	2.673 (2.111)	4.881 (4.369)
IFDI	2.289** (0.723)	2.289*** (0.582)	-0.371 (0.665)	-0.493 (0.657)	0.419 (0.692)
DS	0.172*** (0.027)	0.172*** (0.022)	0.024 (0.034)	0.026 (0.034)	0.179 (0.112)
PGR	-0.565 (1.403)	-0.565 (1.131)	0.995 (1.242)	0.917 (1.242)	2.391* (1.156)
EDUL	53.925*** (8.075)	53.925*** (6.504)	49.144*** (9.685)	50.241*** (9.649)	51.096 (45.817)
RD	-2.398 (2.377)	-2.398 (1.914)	-18.688*** (3.689)	-18.812*** (3.692)	-7.903 (10.473)
EXPT	-0.217 (0.199)	-0.217 (0.161)	-0.213 (0.185)	- -	-0.041 (0.237)
INF	0.597*** (0.052)	0.597*** (0.042)	0.528*** (0.075)	0.521*** (0.075)	0.605*** (0.231)
IND	-0.578** (0.266)	-0.578** (0.214)	-0.339 (0.294)	-0.415 (0.287)	-0.479 (0.499)
Observations	170	170	170	170	170
R-squared	0.742	0.741	0.886	0.886	0.866
Hausman Test (p-value)			95.613 (0.000)	27.047 (0.001)	

*Note*: The symbols *, **, and *** denote statistical significance at the 10%, 5% and 1%, respectively (* *p* < 0.10, ** *p* < 0.05 and *** *p* < 0.01). Parentheses state standard deviation.

To further validate our models, we conduct robustness check by taking out one explanatory variable, EXPT, in the model specification [see Models (4), (9), (14), and (19)]. Our results show the coefficients of OFDI for China (*p < 0*.*05*), Eastern (*p < 0*.*05*), and Central China (*p < 0*.*01*) remain robust, revealing OFDI still has a positive correlation with DI. For Western China, again, the coefficient of OFDI is not statistically significant, demonstrating no significant association between OFDI and DI there.

Apart from performing the traditional panel data regression and robustness check, we employ the GMM procedure to validate our results further. Table 9 presents the results of DI’s persistence, serial correlation, and overidentification tests pertinent to GMM. First, as the DI’s one-year lagged values are positive and significant at a 1% level at the country and regional levels, they capture the dynamic nature of the model. Second, to detect the existence of second-order serial correlation, we find that no serial correlation problem prevailed as p-values for China and its macro-regions exceeded 5%. Lastly, to test whether the instruments are valid, our results show that the p-values of Hansen J-statistic for China, Eastern China, Central China, and Western China are 0.428, 0.334, 0.457, and 0.279, respectively, which fall between 0.25 and 1.00, implying that the independent variables are valid. We conclude that the independent variables are not associated with the error term and are entirely exogenous. Following this, according to the Models (5), (10) and (15), our result demonstrates that the coefficients of OFDI for China (*p < 0*.*05*), Eastern China (*p < 0*.*10*), and Central China (*p < 0*.*05*) remain statistically significant, showing that OFDI still has a positive relationship with DI in those regions. Concomitantly, based on Model (20), the coefficient of OFDI for Western China (*p > 0*.*10*) also indicates that OFDI has no association with DI there.

**Table 9:** DI’s persistence, serial correlation, and overidentification test results for China, Eastern China, Central China, and Western China.

Regions/ Tests	DI’s persistence		Serial correlation estimation			Overidentification estimation		
	Coefficients	Persistence effect	Coefficients	P-values	Existence of second-order serial correlation	Hansen J-statistic	P-values	Validity of all overidentifying restrictions
China	0.704***	Yes	0.007	0.466	No	10.005	0.428	Yes
Eastern China	0.791***	Yes	0.011	0.414	No	3.548	0.334	Yes
Central China	0.899***	Yes	0.004	0.478	No	1.124	0.457	Yes
Western China	0.545***	Yes	0.008	0.458	No	1.387	0.279	Yes

*Note*: The symbol *** represents statistical significance at the 1% level (*** *p* < 0.01).

### 4.4. Outward FDI motivations and provincial DI

Our survey of multinational enterprises’ motivations suggests that those most engaged in OFDI bolster the returns on DI. Table 10 shows the OFDI motivations of the top 100 Chinese multinationals between 2015 and 2018.

**Table 10:** OFDI motivations of the top 100 Chinese multinational enterprises, 2015–2018.

Year	Nature of companies			OFDI motivations															
	SOE	Non-SOE	Total	Resource-seeking				Efficiency-seeking				Market-seeking				Strategic assets-seeking			
				EC	CC	WC	Total	EC	CC	WC	Total	EC	CC	WC	Total	EC	CC	WC	Total
2015	69	30	99	8	3	1	12	4	6	0	10	59	1	0	60	17	0	0	17
2016	65	35	100	7	2	1	9	3	4	0	7	64	1	0	65	17	0	1	18
2017	66	34	100	5	0	2	7	0	6	0	6	63	1	0	64	23	0	0	23
2018	62	38	100	7	1	2	10	5	4	0	9	57	2	0	59	22	0	0	22
**Average**	**66**	**34**	**100**	**7**	**1**	**2**	**10**	**3**	**5**	**0**	**8**	**61**	**1**	**0**	**62**	**20**	**0**	**0**	**20**

*Source*: Outward Foreign Direct Investment Statistics Bulletin of China, 2015–2017, and Report on Development of China’s Outward Investment, 2019.

*Notes*: SOE and non-SOE represent state-owned enterprises and non-state-owned enterprises, and EC, CC, and WC denote Eastern China, Central China, and Western China.

According to Table 10, almost 67% of firms operating in eastern Chinese provinces cited their desire to expand into foreign markets as a motivation for investing outside the region. In comparison, 22% claimed to seek strategic assets, such as advanced technology or technical know-how. Only 8% and 3% of companies in the region publicly cited a search for natural resources and lower-cost production as motivation for engaging in OFDI. Multinational enterprises operating in China’s eastern coastal regions thus invested outside the region to seek new markets and strategic assets. Our regression results suggest these firms’ search for new markets may thus enhance domestic capital formation. Contrasting with previous findings almost two decades ago [23–25], Chinese multinational enterprises may encourage local investment in their home regions as China enters more free trade agreements and expands abroad. Their motivation to develop new markets may gradually develop existing ones provincially. Furthermore, OFDI seeks to bring back competencies for exploitation at home. Instead, these firms search for strategic assets outside their home provinces to boost returns globally. Large fixed investments abroad in these strategic assets provide domestic opportunities for resources.

Multinational enterprises operating in other Chinese provinces have different reasons for engaging in OFDI. First, Eastern China’s multinational enterprises mainly looked for overseas market expansion and strategic assets. On average, sixty-one Chinese multinationals engaged in market-seeking OFDI, while twenty Chinese multinational enterprises committed to strategic asset-seeking OFDI. Second, multinational enterprises operating in Central China claim to invest outside the province to find ways of making the investment at home more profitable. These five companies, on average, engaged in efficiency-seeking OFDI and sought foreign subsidiaries to guarantee the supply of competitive and strategic inputs and explore new and advanced production methods. Finally, the search for natural resources primarily drove OFDI for two Chinese multinationals in Western China. Contrasted with previous findings [24], resource-seeking OFDI positively impacted DI in these provinces. OFDI sought to promote domestic investment in Eastern and Central China by increasing the productivity of such investment.

## 5. Conclusion and policy implications

Our study explores the impact of OFDI on DI in China at the national and regional levels. We conduct the panel regression on a dataset covering ODI and DI in 31 Chinese provinces, municipalities, and autonomous regions from 2005 to 2021. We analyse these data at the national level for China’s three macro-regions and control for province- and time-specific effects. Our findings suggest that OFDI statistically correlates with DI in China, but the relationship varies by region. OFDI correlates with DI in Eastern and Central China but not Western China. Of the four motivations for engaging in such OFDI (efficiency-seeking, resource-seeking, market-seeking, and strategic asset-seeking), the efficiency-seeking, market-seeking, and strategic asset-seeking ones significantly affect domestic capital formation.

Our findings have significant policy ramifications. Many state-owned enterprises in China’s eastern and central provinces play critical roles in investing overseas, stimulating local domestic capital formation. However, for the western part of China, our findings show that simply engaging in more overseas merger and acquisition activities will not necessarily increase investment at home. More DI may not even strengthen local industry or increase profitability at home. Instead, these companies gain their competitive advantage through well-established institutional frameworks, solid state-local relations, and a wealth of financial resources–not from expanding abroad. As different macro-regions have varied exogenous factors, for instance, demands for FDI and manufacturing exports, and endogenous conditions, such as the workers’ levels of education and experience, specific OFDI policies promoting DI’s synergetic effects should be adopted by local governments.

Most importantly, OFDI can improve DI, particularly in Eastern and Central China. Our findings suggest that government policy should foster business activities between domestic state-owned enterprises and private enterprises. Furthermore, the Chinese government’s liberalisation of OFDI rules has the potential to improve DI. Local governments can develop OFDI policies that encourage businesses in their areas to invest in specific strategic industries and local workforce. Specifically, for OFDI policy design, local governments can thoroughly investigate the OFDI motivation embedded in firms. Assume that OFDI is seeking resources as well as strategic assets. In that case, local governments should assist firms in acquiring complementary resources, such as strategic and critical commodities, like rare earth minerals or well-known overseas brand names, by streamlining the process of approving local overseas investment, causing firms to increase their DI by purchasing new advanced fabrication and processing equipment and hiring experienced staff to improve managerial competencies. Next, if the OFDI seeks efficiency, local governments can offer policy incentives to entice firms to invest locally to improve workers’ skills and capabilities, allowing local firms to focus on producing more value-added products. Finally, if local firms seek new markets, the local government can provide tax breaks and rebates and strengthen institutional arrangements with the provincial government to ensure that export regulations are updated in various overseas markets. It can also help firms build more resilient global supply chain networks by implementing significant DI.

Based on these findings, Chinese policymakers could devise new OFDI policies that allow significant regional variations. These policies would maximise the advantages of domestic hierarchical administration while allowing central and local governments to pursue a more balanced regional development within China and its macro-regions. On the other hand, they will play an important role in helping outward-investing firms manage tensions arising from Sino-U.S. geopolitical tensions and the post-COVID-19 economic recovery.

Nonetheless, our study has the following limitations: (1) Because of data limitations, we can only examine the regional variations at the provincial level instead of the prefectural level for further detailed analysis; and (2) The existing levels of data aggregation for three macro-regions may not reveal any intra-regional differences.

Our study quantitatively analysed the association between OFDI and DI at the country and regional levels. In many countries in the Global South, especially for other BRICS countries, examining the OFDI-DI nexus can help produce the regional economic growth of different provinces through local industry-specific and firm-specific OFDI and DI policies. Future research is needed to investigate how OFDI promotes the synergetic effects of DI using qualitative approaches, such as case studies and interviews with firms’ executives and local government officials. This approach may strengthen the theoretical understanding of the existing analytical framework of the OFDI-DI interaction.

## Supporting information

S1 Dataset (XLS)
